# Color: Implications in dentistry

**DOI:** 10.4103/0972-0707.73381

**Published:** 2010

**Authors:** Vimal K Sikri

**Affiliations:** Journal of Conservative Dentistry Editor (1998–2004), Department of Conservative Dentistry and Endodontics, Government Dental College and Hospital, Amritsar, India

**Keywords:** Chroma, hue, shade matching, shade selection, value

## Abstract

The success of restorative dentistry is determined on the basis of functional and esthetic results. To achieve esthetics, four basic determinants are required in sequence; viz., position, contour, texture and color. The knowledge of the concept of color is essential for achieving good esthetics. This review compiles the various aspects of color, its measurements and shade matching in dentistry.

## INTRODUCTION

Restorative dentistry is a blend of science and art. The success of restorative dentistry is determined on the basis of functional and esthetic results. To achieve esthetics, four basic determinants are required in sequence; viz., position, contour, texture and color. Because esthetic dentistry imposes several demands on the artistic abilities of the dentist and the technician, knowledge of the underlying scientific principles of color is essential. Color combination not only improves esthetics but also makes the restoration appear natural and attractive. Continued research on the human visual system has given us greater insight into how color discrimination is affected by environment and other features like disease, drugs and aging. The basic fundamentals of color and light, the radiation spectrum and the optical characteristics of the object is to be understood before evaluating and selecting the proper color shade for the restoration.

## BASIC COLOR SCHEMES

**Figure 1:**
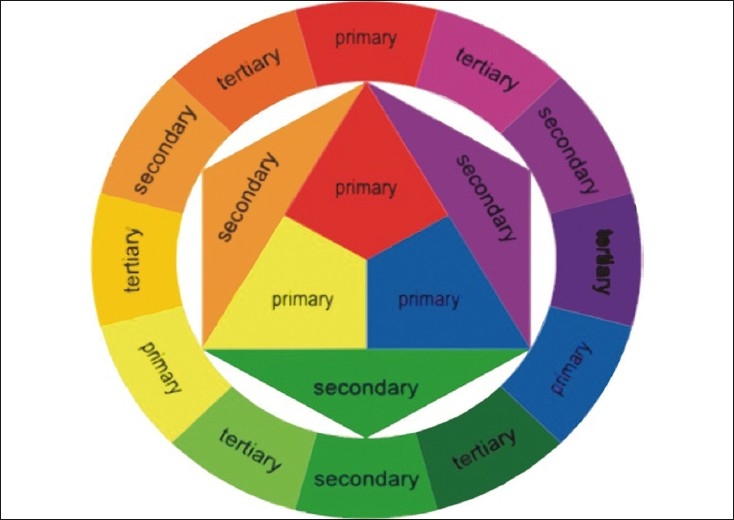
Color wheel

Thecolor wheelorcolor circle [Figure 1] is the basic tool for combining colors. The first circular color diagram was designed by Sir Isaac Newton in 1666. Over the years, many variations of the basic design have been made, but the most common version is a wheel of 12 colors, the primary colorsbeing red, yellow and blue. Three secondary colors (green, orange and purple) are created by mixing two primary colors. Six tertiary colorsare created by mixing the primary and secondary colors.

The color circle can be divided into warm and cool colors.Warm color sare vivid and energetic, and tend to advance in space. Cool colors give an impression of calm, and create a soothing impression. White, black and gray are considered to be neutral.

### Color Harmonies

The color combinations that are considered pleasing are calledcolorharmoniesorcolor chords. They consist of two or more colors having a fixed relation in the color wheel. These are:

- Complementary color scheme (colors that are opposite each other on the color wheel are considered to be complementary colors; e.g., red and green).
- Analogous color scheme (colors that are next to each other on the color wheel).
- Triadic color scheme (three colors that are evenly spaced around the color wheel).
- Tetradic or rectangular color scheme (four colors arranged into two complementary pairs).
- Split complementary color scheme. (In addition to the base color, it uses two colors adjacent to its complement.)
- Square color scheme (similar to the rectangle, but all four colors spaced evenly around the color circle).

### Additive color theory

**Figure 2:**
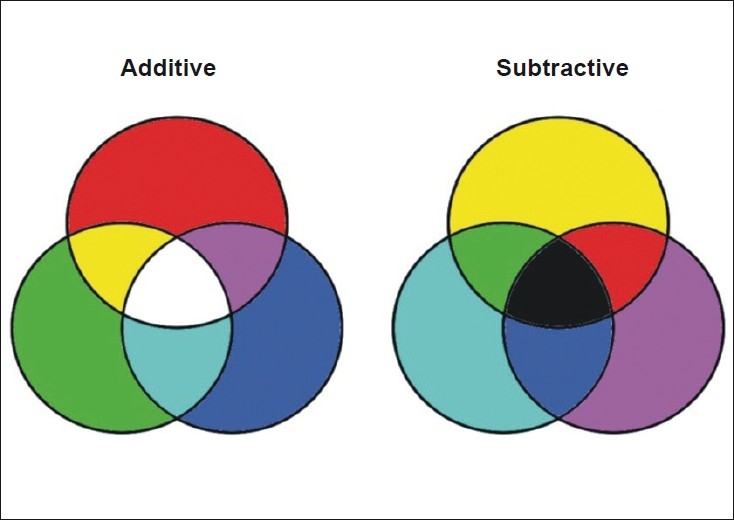
Additive and subtractive colors

The additive primary colors arered, green and blue (RGB). Combining one of these additive primary colors with equal amounts of another one results in the additive secondary colors of cyan, magenta and yellow [Figure 2]. Combining all three additive primary colors in equal amounts will produce the color white. Remember, combining additive colors creates lighter colors. Therefore, adding all three primary colors results in a color so “light” that it is actually seen as white.

**Table:** Additive colors combined in equal parts

Blue + green	=	Cyan
Red + blue	=	Magenta
Green + red	=	Yellow
Red + green + blue	=	White

**Table:** Additive colors combined in unequal parts

1 Green + 2 red	=	Orange
1 Red + 2 green	=	Lime
1 Green + 1 blue + 4 red	=	Brown

On changing the brightness of each of the three primary colors by varying degrees, one can make a wide range of colors.

### Subtractive color theory

The subtractive primary colors arecyan, magenta and yellow [Figure 2]. Subtractive color mixing occurs when light is reflected off a surface or is filtered through a translucent object. For example, a red surface only appears red because itabsorbs (subtracts)all of the light that is not red and onlyreflects or allowsthe red light.

### Subtractive colors mixing

**Table:** 

Combine	Absorbs	Leaves
Cyan + magenta	Red + green	Blue
Cyan + yellow	Red + blue	Green
Magenta + yellow	Green + blue	Red
Cyan + magenta + yellow	Red + green + blue	Black

Mixing of two subtractive primary colors result in a color that is complimentary to the remaining primary. For example, if cyan and magenta are mixed, they will form blue, which is complimentary to yellow (third subtractive primary color).

### Nature of color

Color is all about light. For color to be seen, light is reflected from an object and stimulates the neural sensors in the eye’s retina to send a signal that is interpreted in the visual cortex of the brain.[1] The reflected components of incident white light determine the color of an object. Transparent materials allow for the passage of light with little change. Translucent materials scatter, transmit and absorb light. Opaque materials reflect and absorb; however, they do not transmit. Most of the color found in the natural tooth is established within the tooth. The semitranslucent structure of tooth makes the color-matching procedure more complex when compared with an opaque object. Surface characteristics, such as gloss, curvature and texture, affect the degree of light diffusion when striking a particular object.[2]

### Light

Light is electromagnetic radiation that can be detected by the human eye. Natural white light falls between 380 and 770 nm along the electromagnetic spectrum, having a couple of component bands along the spectrum. The component bands produce six different sensations, i.e. red, orange, yellow, green, blue and violet. However, there is an infinite number of gradations among the component bands with ill-defined boundaries. The color of any object is dependent on the illuminant in which it is viewed. If incident light does not contain a particular wavelength segment, the object cannot reflect it. Colorants (pigments or dyes) are responsible for chromatic reflection of light.[1] The chemical composition of a colorant selectively absorbs one part of the visible spectrum more than another. When a particular wavelength segment of light is reflected and enters the eye, the sensation of color is produced.

### Perception of color

As light enters the eye through the cornea and lens, an image is focused on the retina. The amount of light entering the eye is controlled by the iris, which dilates or constricts depending on the level of illumination. The retinal rods and cons can adjust the variation of light intensity. The area around the fovea centralis has a mixture of sensors responsible for differences in color discrimination among observers with normal color vision.[3] The accuracy of color perception depends on the area of the retinal field stimulated by light. In high illumination, the pupil narrows and when light is dim, the pupil widens, stimulating sensors that are less accurate. As a regulator of pupil diameter, light intensity is a critical factor in color perception and shade matching. The three important features that reflect color matching are successive contrast, simultaneous contrast and color constancy.[1] Successive contrast is the projection-negative effect that occurs after staring at a colored object. Simultaneous contrast is an instantaneous change in chromatic sensitivity, characterized by a change in appearance due to the surrounding colors. Color constancy occurs because we perceive certain objects as being of different color and the object seems to be of the same color even if the light received by the eye varies.[4] A neural response is involved in color vision and constant stimulation by a single color may result in color fatigue and decrease in the eye’s response. Our ability to perceive color and visual acuity is also affected by aging, chronic illnesses, glaucoma and medications like oral contraceptives, ibuprofen, antiepileptic drugs, aspirin and antibiotics and lidocaine, etc.

## EFFECT OF THE SURROUNDINGS

Color perception is affected by the reflection or interference from the surrounding colors. The effects of clothing and make-up, especially lipstick, should be neutralized. One should stare at a tooth for less than 5 s because our eyes become accommodated to the red and yellow colors.[4] The after-image that occurs when looking continuously at an object of one color can be minimized by looking at a blue object between assessing different shade tabs. Blue backgrounds, however, are not appropriate because they also cause after-images and may bias your perception to its complementary color “orange.” The eyes should be given a break with a neutral grey background such as a Pensler Shield (Kulzer), which is designed to screen out the background color glare.

### Light quality

The quality of light source is the most influential factor whendetermining tooth shade. The ideal light source is natural light, occurring around mid-day for accurate color comparison. The time of the day, month and weather conditions affect the color of sunlight. If the light source changes, then the light reflected from an object changes too; in that case, a different color is perceived. The absence of ideal conditions has led to the use of artificial lighting for color matching. The light source that approximates standard daylight is ideal for shade matching. Color temperature, spectral reflectance curves and Color Rendering Index (CRI) are all used to measure the capacity to reproduce standard daylight (CRI over 90 is recommended for color matching). Dental unit lights are usually incandescent lights that emit light high in the red–yellow spectrum and are low at the blue end. Regular cool white fluorescent lights are high in the green–yellow spectrum. Color-corrected fluorescent lights are also available, which render the color more accurately.[3] Full-spectrum light-emitting diodes (LEDs) are now replacing incandescent bulbs. The shade-matching ability is better with a light-correcting source than under natural light.[5] A new device that eliminates the variability of different light sources, “The Optilume Trueshade,” uses full-spectrum LEDs and shows a color spectrum similar to mid-day light. Diffusion lenses over the LEDs mix the three (RGB) colors of light emitted by the individual color diodes to create optimum, diffuse daylight. With the LEDs set at a 45-degree angle to minimize spectral reflectance or glare, the clinician can more accurately assess the true color.[4] A unique feature of Optilume Trueshade is the ability to reduce the intensity of the light source while maintaining the color temperature. A lower-intensity light allows for better perception of surface details, such as topography, ridges and enamel striations.

### Three dimensions of color

Color is usually described according to the Munsell color space in terms of hue, value, and chroma. Hue is the attribute of a color that enables the clinician to distinguish between different families of color, whereas value indicates the lightness of a color. Chroma is the degree of color saturation. When color is determined using the Munsell system, value is determined first followed by chroma. Hue is determined last by matching with shade tabs of the value and chroma already determined.[6]

### Hue

**Figure 3:**
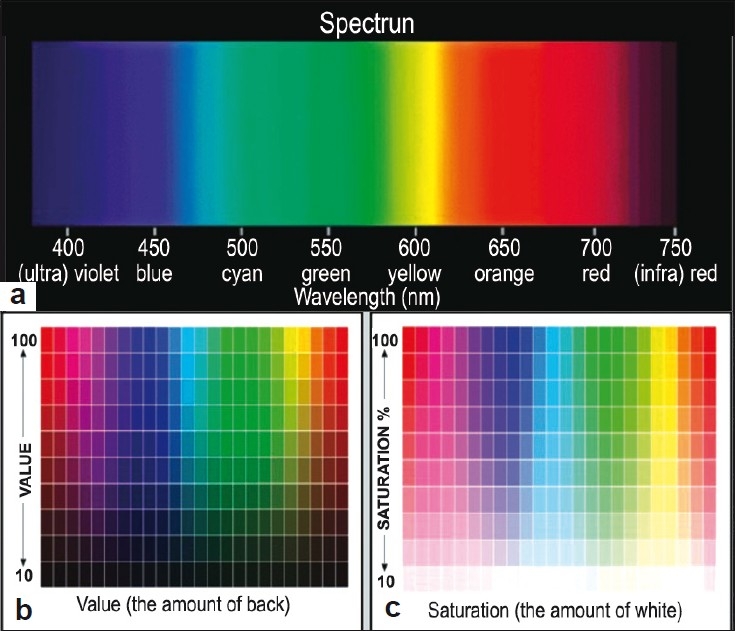
Three dimensions of color a) Hue b) Value c) Chroma

“Hue” is the quality that distinguishes one family of color from another. It is specified as the dominant range of wavelengths in the visible spectrum that yields the perceived color, even though the exact wavelength of the perceived color may not be present [Figure 3a]. Hue is a physiologic and psychologicinterpretation of a sum of wavelengths. Hue is represented by A, B, C or D on the commonly used Vita Classic shade guide.[4]

### Value

“Value,” or brightness, is the amount of light returned from an object [Figure 3b]. Munsell described value as a white-to-black gray scale. Bright objects have lower amounts of gray and low-value objects have larger amounts of gray and will appear darker.[4] The brightness of a crown is usually increased in two ways: by lowering chroma or by increasing the reflectivity of the surface. Lowering value means less light returns from the illuminated object and the remaining light is being absorbed or scattered elsewhere.

### Chroma

“Chroma” is the saturation, intensity or strength of the Hue [Figure 3c].[4] If any dye (say red) is added into a glass of water and the same dye is added again and again, the intensity increases, but the color remains the same (hue). As more dye is added, the mixture appears darker; thus, the increase in chroma has a corresponding change in value. As chroma is increased, the value is decreased; chroma and value are inversely related. Higher numbers on the Vita Classic shade guide represent increased chroma.[6]

### Translucency

Human teeth are characterized by varying degrees of translucency, which can be defined as the gradient between transparent and opaque. Generally, increasing the translucency of a crown lowers its value because less light returns to the eye. With increased translucency, light is able to pass the surface and is scattered within the restoration. The translucency of enamel varies with the angle of incidence, surface texture and luster, wavelength and level of dehydration.[7]

### Fluorescence

Fluorescence is the absorption of light by a material and the spontaneous emission of light in a longer wavelength. In a natural tooth, it primarily occurs in the dentin because of the higher amount of organic material. Ambient near-UV light is absorbed and fluoresced back as light primarily in the blue end of the spectrum; however, it occurs at all wavelengths. The more the dentin fluoresces, the lower the chroma.[7] Fluorescent powders are added to crowns to increase the quantity of light returned back to the viewer, block out discolorations and decrease chroma. This is especially beneficial in high-value shades as it can raise value without negatively affecting translucency when placed within the dentin porcelain layers.

### Opalescence

Opalescence is the phenomenon in which a material appears to be of one color when light is reflected from it and of another color when light is transmitted through it. A natural opal is an aqueous disilicate that breaks transilluminated light into its component spectrum by refraction. Opals act like prisms and refract different wavelengths to varying degrees.[7] The shorter wavelengths refract more and require a higher critical angle to escape an optically dense material than the reds and yellows. The hydroxyapatite crystals of enamel also act as prisms. Wavelengths of light have different degrees of translucency through teeth and dental materials. When illuminated, opals and enamel will transilluminate the reds and scatter the blues within their body; thus, enamel appears bluish even though it is colorless. The opalescent effects of enamel brighten the tooth and give it optical depth and vitality.

### Metamerism

Two colors that appear to match under a given lighting condition but have different spectral reflectance are called metamers, and the phenomenon is known as metamerism. The problem of metamerism can be avoided by selecting a shade and confirming it under different lighting conditions, such as natural daylight and fluorescent light.[8]

## MEASUREMENT OF COLOR

Color determination in dentistry can be divided into two categories:

- Visual
- Instrumental

### (A) Visual technique

**Figure 4:**
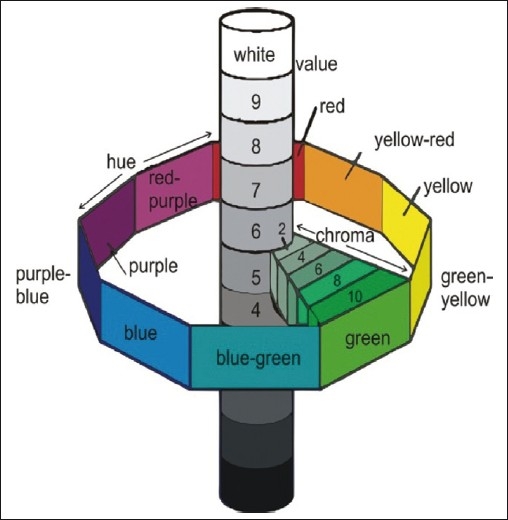
Munsell color system

A popular system for visual determination of color is the Munsell color system, the parameters of which are represented in three dimensions [Figure 4]. Value (lightness) is determined first by the selection of a tab that most nearly corresponds with the lightness or darkness of the color.[9] Value ranges from white (10/) to black (0/). Chroma is determined next with tabs that are close to the measured value but are of increasing saturation of color. Chroma ranges from achromatic or gray (/0) to a highly saturated color (/18). Hue is determined last by matching with color tabs of the “value” and “chroma” already determined. Hue is measured on a scale from 2.5 to 10 in increments of 2.5 for each of the 10 color families (red, R; yellow-red, YR; yellow, Y; green-yellow, GY; green, G; blue-green, BG; blue, B; purple-blue, PB; purple, P; red-purple, RP).

Visual color determination of a patient’s tooth is the most frequently applied method in clinical dentistry. However, visual determination of shade selection has been found to be unreliable and inconsistent. Visual color assessment is dependent on the observer’s physiologic and psychologic responses to radiant energy stimulation. Inconsistencies may result fromuncontrolled factors such as fatigue, aging, emotions, lighting conditions, previous eye exposure, object and illuminant position and metamerism. Paravina (2002)[10] evaluated a newly developed visual shade-matching apparatus, Shademat Visual+ (SV+). The SV+ apparatus enabled better shade-matching results than daylight. Correlated color temperature of the daylight varied from 4,500 to 6,800 K, while light intensity varied from 140 to 1,000 lux. In SV+ trials, these values were constant at the measuring place: 5,000 K and 1,400 lux. There is need for a more scientific and consistent means of shade matching in restorative dentistry.

### (B) Instrumental technique

**Figure 5:**
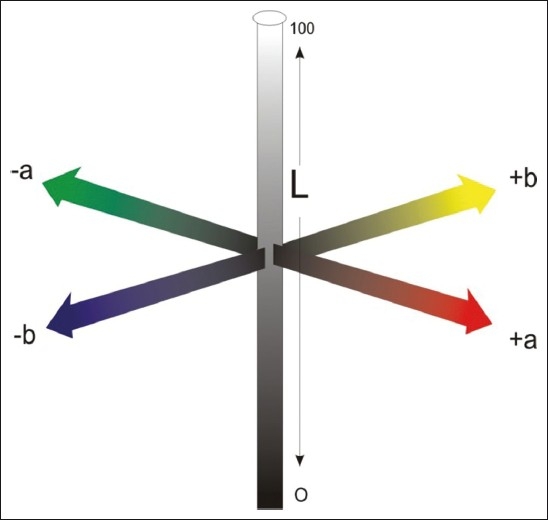
L* a* b* system

In this system, the color space consists of three coordinates: L*, a* and b* [Figure 5].[9] The L* refers to the lightness coordinate, and its value ranges from 0 for perfect black to 100 for perfect white. The a* and b* are the chromaticity coordinates in the red–green axis and yellow–blue axis, respectively. Positive a* values reflect the red color range and negative values indicate the green color range. Similarly, positive b* values indicate the yellow color range while negative values indicate the blue color range. The differences in the lightness and chromaticity coordinates (∆L*, ∆a*, ∆b*) as a result of UV light exposure are determined first, and the total color change (∆E*_ab_) can be calculated using the relationship

$${\mathrm{∆E}}_{\mathrm{ab}}^{*}\;=\;{\left({\mathrm{∆L}}^{*2}\;+\;{\mathrm{∆a}}^{*2}\;+\;{\mathrm{∆b}}^{*2}\right)}^{1/2}$$

Instrumental color analysis, on the other hand, offers a potential advantage over visual color determination because instrumental readings are objective, can be quantified and are more rapidly obtained. Spectrophotometers and colorimeters have been used with modifications in an attempt to overcome problems with visual shade matching in dentistry. Photoelectric tristimulus colorimeters have the potential to remove some of the shortcomings of the visual method, and have been shown to provide accurate and repeatable measurements; however, they are not error-proof. In dentistry, the results of a colorimetric device can be altered because the standardized illuminating light emitted from the device may be scattered, absorbed, transmitted, reflected and even displaced in a sideways direction as a result of the translucent optical properties of teeth and dental ceramics. Haywood *et al*. (1994) found out that colorimeters are designed for flat surfaces rather than the curved translucent surfaces found on teeth.

The non-uniformcolor properties of teeth involve a complex layering of tooth structure and subtle color changes that challenge even the best instruments. Additionally, the high cost and limited utility of these instruments prevent their use in clinical dental practice.

## SHADE-TAKING DEVICES

These devices have been designed to aid clinicians and technicians in the specification and control of tooth color. The earliest color-measuring devicedesigned for clinical use was a “filter colorimeter.” The “Chromascan,” introduced in the early 80s, enjoyed limited success due to its inadequate design and accuracy. Because esthetics is a major focus of dental marketing coupled with the availability of improved color-measuring optics, newer devices are being planned to overcome the challenge of shade matching. Color-measuring devices usually consist of a detector, signal conditioner and software that process the signal in a manner that makes the data usable in the dental operatory or laboratory.

### Colorimeters

**Figure 6:**
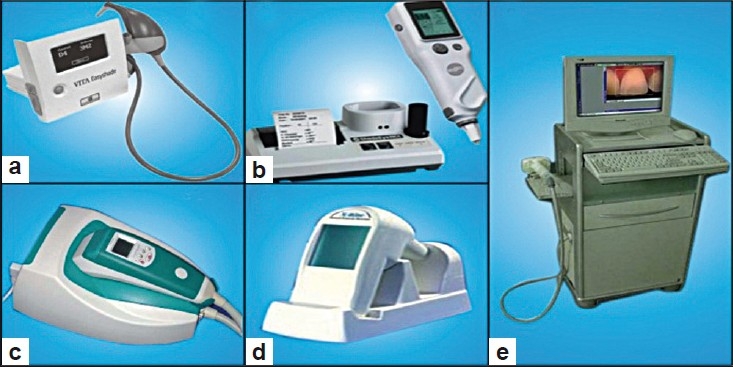
Shade taking devices a) Vita easyshade b) Shade eye NCC c) Shade scan d) Shade rite dental vision system e) Spectroshade

Filter colorimeters generally use three or four silicon photodiodes that have spectral correction filters. These filters act as analog function generators that limit the spectral characteristics of the light striking the detector surface.[1] The filter colorimeters are considered inferior to scanning devices such as spectrophotometers and spectroradiometers because of the inability to match the standard observer functions. However, because of their consistent and rapid sensing nature, these devices can be used for quality control. ShadeEye is an example of a colorimeter [Figure 6] based on the natural color concept.

## DIGITAL CAMERAS AS FILTER COLORIMETERS

The digital camera technology is also being used for shade matching. Instead of focusing light on the film to create a chemical reaction, digital cameras capture images using charged coupled devices (CCDs), which contain millions of microscopically small light-sensitive elements. Like photodiodes, each photosite responds only to the total light intensity that strikes its surface. To get a full color image, most sensors use filtering to look at the light in its three primary colors in a manner analogous to the filtered colorimeter. There are several ways of recording the three colors in a digital camera.[11] The highest-quality cameras use three separate sensors, each with a different filter over it. Light is directed to the different filter/sensor combinations by placing a beam splitter in the camera. The beam splitter allows each detector to see the image simultaneously. The advantage of this method is that the camera records each of the three colors at each pixel location. The ShadeRite Dental Vision System [Figure 6] and ShadeScan [Figure 6] combine digital color analysis with colorimetric analysis, but SpectroShade [Figure 6] is the only one that combines digital color imaging with spectrophotometric analysis.

## SPECTROPHOTOMETERS AND SPECTRORADIOMETERS

Spectrophotometers and spectroradiometers are instruments designed to produce the most accurate color measurements. Spectrophotometers differ from spectroradiometers primarily because they include a stable light source. There are two types of basic designs commonly used for these instruments. The traditional scanning instrument consists of a single photodiode detector that records the amount of light at each wavelength.[2] The light is divided into small wavelength intervals by passing it through a monochromator. A more recent design uses a diode array with a dedicated element for each wavelength. This design allows for the simultaneous integration of all wavelengths. Both designs are considerably slower than filter colorimeters. However, these arethe routinely used color-measuring devices. Vita EasyShade is an example of a spectrophotometer [Figure 6].

### Shade guides

**Figure 7:**
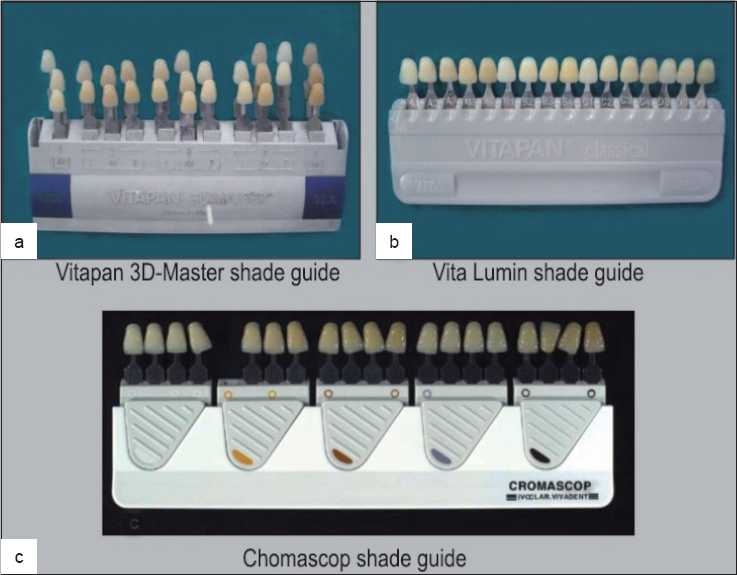
Shade a) Vita 3D-Master shade guide b) Vita classical shade guide c) Chromascop shade guide

Early shade guides were derived from tooth colors that were considered pleasing rather than from the distribution of shades found in the general population. Clark introduced a custom shade guide in 1931 based on visual assessment of human teeth, recorded in Munsell Hue, Value and Chroma. Acknowledging the deficiencies of the available guides, Sproull, in the early 70s, suggested that an ideal shade guide should consist of shade (color) tabs that are well distributed and logically arranged in color space, preferably based on the Munsell color system. Quality control issues regarding color mismatches of shade tab and porcelain batches from the same manufacturer could be as problematic as mismatches among manufacturers. In the mid 90s, Miller acknowledged that the material of the shade guide should be the same as the restoration, and the thickness of the shade guides should not be more than the average porcelain veneer. The limitations of shade guides are factors that compromise shade-matching procedures in dentistry and contribute to the dissatisfaction of clinicians, technicians and patients. A new generation of shade guides has been developed to address these deficiencies. Shofu offered the Natural Color Concept[12] while Vita introduced a 3-dimensional shade guide system (Vita 3D-Master). The Natural Color Concept[13] system consists of 208 color blends based on 38 basic shades. The manufacturer purports that these blends are logically arranged in L*a*b* color space according to Munsell Hue, Chroma and Value. In addition, the shade guides and veneering material are made of the same material to avoid the effect of metamerism. The Vita 3D-Master shade guide[1] [Figure 7a] features a systematic colorimetric distribution of 26 shade tabs within the tooth color space. The manufacturer purports that this shade guide demonstrates an equidistant distribution in the color space. The shade guide is organized into five primary value levels, with a secondary distribution based on chroma and hue. These value groups are arranged from the lightest (value level 1) to the darkest (value level 5), left to right. Intermediate shades can be achieved based on mixing formulas. The manufacturer advocates a three-step process: value is determined first in making a shade determination and then the chroma and hue are determined. The selection process is simplified because the number of choices decreases throughout the procedure. The shade tabs arrangement in the Vita Classical[1] [Figure 7b] is by hue where as in the Chromascop guides[1] [Figure 7c], the tabs are arranged in five clearly discernible value levels. Within each level are tabs that represent different chromas and hues. The five levels cover that area of the CIELAB color solid occupied by natural teeth. The lightest value level has only two chroma steps of single hue, and the darkest value level has three chroma steps of one hue. Groups 2, 3 and 4 have three chroma levels of the middle and orange hue and two chroma levels in each hue shift toward yellow or red. The sequence of shade selection is value, then chroma, followed by hue.

### Shade selection

There are certain principles that should be followed during shade selection.[14–20] These are as follows:

1. The patient should be viewed at the eye level so that the most color-sensitive part of the retina will be used.
2. Shade comparison should be made under different lighting conditions. Normally, the patient is taken to a window and the color is confirmed in natural daylight after initial selection under incandescent and fluorescent lightening.
3. The teeth to be matched should be clean.
4. Shade comparison should be made at the start of the patient visit.
5. Brightly colored clothing should be draped and lipstick should be removed.
6. Shade comparison should be made quickly, with the color samples placed under the lip directly next to the tooth being matched.
7. The eye should be rested by focusing on a gray-blue surface immediately before a comparison since this balances all the color sensors of the retina and resensitizes the eye to the yellow color of the tooth.

Dagg *et al*. (2004)[15] investigated some of the factors on which accurate shade taking depends. Four main factors were investigated, namely, the difference between the two types of porcelain used, the effect of light quality, the effect of porcelain thickness and the experience of the observer. These results indicate that the most influential factor on shade taking was the light quality.
